# Impact of transcranial Doppler sonography for detecting ischemic stroke: A protocol of systematic review and meta-analysis

**DOI:** 10.1097/MD.0000000000020451

**Published:** 2020-05-29

**Authors:** Wen-Juan Liu, Ya-Juan Zhang

**Affiliations:** Department of Neurology, First Affiliated Hospital of Jiamusi University, Jiamusi, China.

**Keywords:** ischemic stroke, sensitivity, specificity, transcranial doppler sonography

## Abstract

**Background:**

This study aims to explore the impact of transcranial Doppler sonography (TDS) for detecting ischemic stroke (IS).

**Methods:**

PUBMED, EMBASE, Cochrane Library, PsycINFO, Cumulative Index to Nursing and Allied Health Literature, Allied and Complementary Medicine Database, WANGFANG, Chinese Biomedical Literature Database, and China National Knowledge In-frastructure will be utilized to examine case-controlled studies that used TDS for detecting IS. All electronic databases will be searched from inception to March 20, 2020. All study selection, data extraction, and study quality assessment will be carried out by 2 independent reviewers. All study quality will be assessed by Quality Assessment of Diagnostic Accuracy Studies tool, and statistical analysis will be performed by RevMan V.5.3 software and Stata V.12.0 software.

**Results:**

This study will explore the impact of TDS for detecting IS through sensitivity, specificity, positive and negative likelihood ratio, and diagnostic odds ratio.

**Conclusion:**

: This study expects to find out whether TDS can be utilized for IS detection.

Systematic review registration: INPLASY202040155.

## 1 Introduction

Ischemic stroke (IS) is the most subtype of stroke, and accounts for 68% of all strokes.^[1–4]^ It is a leading cause of mortality and disability around the world despite significant scientific and therapeutic advances.^[5–8]^ Thus, it is a critical need to enhance stroke prevention and treatment based on its diagnosis and detection at early stage.^[9–12]^ Many researchers have studied the impact of transcranial Doppler sonography (TDS) in the detection of IS.^[13–20]^ However, no systematic review draws a definite conclusion of this issue. This study will be performed to explore the impact of TDS for detecting IS.

## 2 Methods

### 2.1 Objective

This study aims to explore the impact of TDS for detecting IS.

### 2.2 Study registration

This study has been registered on INPLASY202040155, and has been reported according to the guideline of Preferred Reporting Items for Systematic Reviews and Meta-Analysis (PRISMA) Protocol statement.^[21]^

### 2.3 Eligibility criteria for study selection

#### 2.3.1 Type of studies

This study will include all potential case-controlled studies (CCSs) that investigate the impact of TDS for detecting IS. However, this study will not include animal studies, reviews, case studies, and non-CCSs.

#### 2.3.2 Type of participants

All patients who were diagnosed as having IS will be included in this study, irrespective their race, age, and severity of IS.

#### 2.3.3 Type of index test

Index test: Any form of TDS for detection on patients with IS will be included in this study.

Reference test: All patients with computerized tomography or magnetic resonance imaging-proven IS will be included in the control group.

#### 2.3.4 Outcome measurements

This study comprises of sensitivity, specificity, positive likelihood ratio, negative likelihood ratio, and diagnostic odds ratio.

### 2.4 Data sources and search strategy

#### 2.4.1 Electronic searches

The following electronic databases will be searched: PUBMED, EMBASE, Cochrane Library, PsycINFO, Cumulative Index to Nursing and Allied Health Literature, Allied and Complementary Medicine Database, WANGFANG, Chinese Biomedical Literature Database, and China National Knowledge In-frastructure from inception to March 20, 2020 with no limitations of language and publication status. All potential CCSs that examined the impact of TDS for detecting IS will be included. We will build search strategy sample for PUBMED in Table 1. We will adapt similar search strategies for other electronic databases.

**Table 1:**
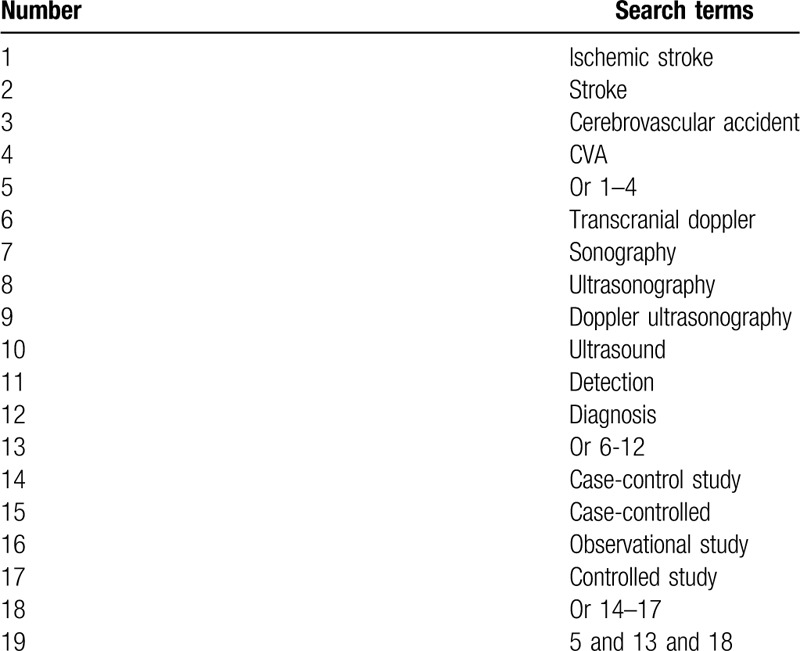
Search strategy applied in PubMed.

#### 2.4.2 Other resources

We will identify conference abstracts, websites of clinical registry, and reference lists of associated reviews.

### 2.5 Data collection and analysis

#### 2.5.1 Selection of studies

All searched studies will be imported to Endnote 7.0 software, and all duplications and irreverent studies will be eliminated. Full articles of all potential studies will be examined against all inclusion criteria. We will summarize reasons for all excluded studies. The process of study selection will be presented in a PRISMA flow chart.

#### 2.5.2 Data collection and management

Two independent reviewers will independently collect data utilizing a standardized data extraction sheet. If there will be divergences between 2 reviewers, they will be settled by a third reviewer through discussion. We will collect data of study identification details (such as title, first author, and time of publication), trial design, trial setting, population characteristics (such as age, sex, and diagnostic criteria), and details of index and reference tests, outcomes, results, and conclusions.

#### 2.5.3 Dealing with missing data

If we identify any unclear or missing data, we will contact primary authors to inquire them. We will analyze available data using intention-to-treat analysis if we cannot obtain those data.

### 2.6 Methodological quality assessment

Two reviewers will independently assess study quality using Quality Assessment of Diagnostic Accuracy Studies (QUADAS-2) tool through 4 domains.^[22]^ If there are any conflicts between 2 reviewers, we will invite a third reviewer to solve them by discussion.

### 2.7 Statistical analysis

This study will apply RevMan V.5.3 software and Stata V.12.0 software to pool the data and to perform statistical analysis. We will use Stata V.12.0 software to estimate sensitivity, specificity, positive likelihood ratio, negative likelihood ratio, and diagnostic odds ratio by using 2 × 2 tables of primary studies. We will estimate outcome data using descriptive statistics and 95% confidence intervals. We will also carry out a descriptive forest plot, and a summary receiver-operating characteristic plot.

#### 2.7.1 Assessment of heterogeneity

We will check statistical heterogeneity across studies by *I*^*2*^ statistic. *I*^2^ ≤50% suggests reasonable heterogeneity, and *I*^*2*^ > 50% reveals obvious heterogeneity.

#### 2.7.2 Data synthesis

If *I*^2^ ≤50%, we will pool the data and will carry out meta-analysis. If *I*^*2*^ > 50%, we will perform a subgroup analysis and will synthesize data based on the results of subgroup analysis. If we still find considerable heterogeneity after subgroup analysis, we will not pool the data, and meta-analysis will be not undertaken. We will use a bivariate random-effects regression approach to estimate sensitivity and specificity.

#### 2.7.3 Subgroup analysis

We will carry out a subgroup analysis to explore remarkable heterogeneity based on different study and patient characteristics, and index and reference tests.

#### 2.7.4 Sensitivity analysis

We will perform a sensitivity analysis to examine the stability of outcome results by deleting low quality studies.

#### 2.7.5 Reporting bias

We will perform funnel plots and associated regression tests to examine if there are any reporting biases.^[23]^

### 2.8 Ethics and dissemination

This study will not need research ethic approval because no individual patient data will be collected. We expect to publish this study on a peer-reviewed journal.

## 3 Discussion

IS is one of the most common neurological diseases globally. If it is not treated satisfactorily, it will cause poor quality of life in patients with IS. Thus, it is very important to diagnose this condition at early stage. Although most previous studies have shown that TDS can help detect IS, all conclusions have reached based on the basis of independent study, and no systematic review has studied this issue. Thus, this study will systematically assess the impact of TDS for IS detection. It will summarize the most recent evidence to help judge whether TDS can be used for detecting IS.

## Author contributions

**Conceptualization:** Wen-juan Liu, Ya-juan Zhang.

**Data curation:** Wen-juan Liu, Ya-juan Zhang.

**Formal analysis:** Wen-juan Liu.

**Funding acquisition:** Ya-juan Zhang.

**Investigation:** Ya-juan Zhang.

**Methodology:** Wen-juan Liu, Ya-juan Zhang.

**Project administration:** Ya-juan Zhang.

**Resources:** Wen-juan Liu.

**Software:** Wen-juan Liu.

**Supervision:** Ya-juan Zhang.

**Validation:** Wen-juan Liu, Ya-juan Zhang.

**Visualization:** Wen-juan Liu, Ya-juan Zhang.

**Writing – original draft:** Wen-juan Liu, Ya-juan Zhang.

**Writing – review & editing:** Wen-juan Liu, Ya-juan Zhang.
